# UICD: A new dataset and approach for urdu image captioning

**DOI:** 10.1371/journal.pone.0320701

**Published:** 2025-06-02

**Authors:** Rimsha Muzaffar, Syed Yasser Arafat, Junaid Rashid, Jungeun Kim, Usman Naseem

**Affiliations:** 1 Department of CS&IT, Mirpur University of Science and Technology (MUST), Mirpur-10250 (AJK), Pakistan; 2 Department of Artificial Intelligence and Data Science, Sejong University, Seoul, Republic of Korea; 3 Department of Computer Science and Engineering, Inha University, Incheon, Republic of Korea; 4 School of Computing, Macquarie University, Sydney, Australia; Government College University Faisalabad, PAKISTAN

## Abstract

Advancements in deep learning have revolutionized numerous real-world applications, including image recognition, visual question answering, and image captioning. Among these, image captioning has emerged as a critical area of research, with substantial progress achieved in Arabic, Chinese, Uyghur, Hindi, and predominantly English. However, despite Urdu being a morphologically rich and widely spoken language, research in Urdu image captioning remains underexplored due to a lack of resources. This study creates a new Urdu Image Captioning Dataset (UCID) called UC-23-RY to fill in the gaps in Urdu image captioning. The Flickr30k dataset inspired the 159,816 Urdu captions in the dataset. Additionally, it suggests deep learning architectures designed especially for Urdu image captioning, including NASNetLarge-LSTM and ResNet-50-LSTM. The NASNetLarge-LSTM and ResNet-50-LSTM models achieved notable BLEU-1 scores of 0.86 and 0.84 respectively, as demonstrated through evaluation in this study accessing the model’s impact on caption quality. Additionally, it provides useful datasets and shows how well-suited sophisticated deep learning models are for improving automatic Urdu image captioning.

## Introduction

**Fig 1:**
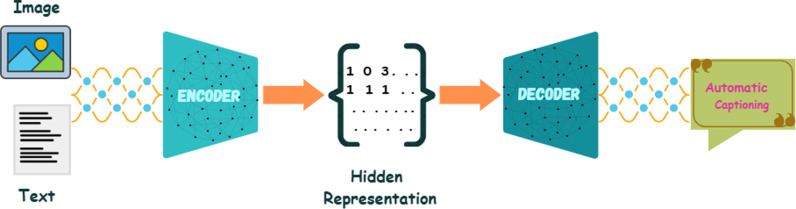
The generic architecture for automatic captioning illustrates how the encoder merges images and vectors, and how the decoder generates sequence predictions.

At the intersection of natural language processing and computer vision is the dynamic field of image captioning, which focuses on generating descriptive text for images. Computer vision involves using algorithms and models to interpret and analyze visual information, transforming it into meaningful data. This field encompasses various techniques such as vehicle identification [1], text segmentation [2], text recognition [3–4], and human detection [5], which is crucial for extracting detailed features from images. These techniques contribute to the process of converting visual content into vector representations. Following this, image captioning uses a natural language processing (NLP) model to decode these vectors and generate textual descriptions of the images, as illustrated in Fig 1. It involves training a machine to look at images and generate textual descriptions of their content, just as humans do. This approach ensures that the captions accurately reflect the content and context of the images.

**Fig 2:**
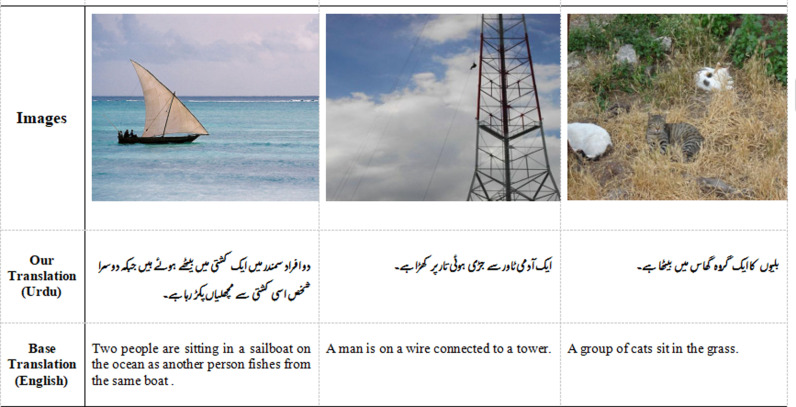
Captions from Flickr30k dataset in English 3^rd^ row and Urdu 2^nd^ row (ours).

This emerging field has seen significant research efforts tailored to different regional languages like Arabic[6], Chinese [7], Uyghur [8], Hindi [9], and English [10–13]. The researchers in the field of these languages developed their corpus or datasets in their regional languages, either from scratch or from existing datasets like Flickr8k [12], Flickr30k [14], MSCOCO [10]. Most research in this field has focused on English, with limited attention to other languages like Urdu. To bridge this gap, our work develops models specifically for Urdu, addressing its unique linguistic challenges. A sample from Flickr 30k is shown in Fig 2.

Urdu is the national language of Pakistan, and it is widely spoken and understood in Pakistan, India, Nepal, and Bangladesh. According to the 2022 edition of Ethnologue [16], with almost 230 million speakers, Urdu is the tenth most spoken language in the world. Additionally, speakers of Urdu can be found in the Middle East, Europe, India, Australia, and the United States. The Urdu script employs the Nastaliq style, a calligraphic writing system derived from Arabic and Persian scripts, known for its distinct aesthetic and flowing structure. Linguistically, Urdu follows the abjad system, where consonants and long vowels are explicitly written, while short vowels are typically omitted. Nastaliq is a visual representation style, while abjad refers to the phonetic writing system. This combination impacts tokenization and preprocessing NLP, as the omission of short vowels can lead to ambiguities. Additionally, Urdu’s bidirectional nature writes numbers left-to-right and text right-to-left, with letters changing shape based on their position, like initial, medial, final, or isolated. These features highlight the script’s unique linguistic and structural complexity [17]. Understanding these linguistic features is essential for developing robust preprocessing methods and ensuring that the automatic translation and captioning processes respect the nuances of the Urdu language.

The BLEU Score is a widely used metric for evaluating caption generation models. It evaluates the quality of generated captions by comparing them to reference captions using BLEU-1, BLEU-2, BLEU-3, and BLEU-4 scores [18–19]. This technology has numerous potential applications, including making images and videos more accessible to people with visual impairment [20], improving image search and retrieval [21], enhancing social media [22] and e-commerce platforms [23]. Current developments in deep learning [24], particularly the use of Convolutional Neural Networks (CNN) [25] and Recurrent Neural Networks (RNNs) [26], have produced notable advancements in the precision and fluency of machine-generated automatic image captioning. However, the challenges of generating captions that are diverse, creative, and contextually relevant remain. Researchers are actively working to develop more sophisticated models that can enhance comprehension of the visual contents of images and generate captions that are not only accurate but also compelling and engaging. This paper emphasizes that it is necessary to expand resources and solutions for Urdu to promote research in the language, especially in the area of captions for images, which has become more well-known recently. Table 1 illustrates the most recent developments in Urdu image captioning, with the majority of the automatic captioning performed by researchers using the Flickr8k dataset containing 8k images. Our study is the first to use 30k images for the image captioning system in Urdu.

**Table 1:** Comparison of Urdu Image Captioning Studies.

Name	Inspire Dataset	Actual Images	Author/Year	Language	Images	Captions
Efficient Urdu Caption Generation using Attention-based LSTM [27]	Flickr8k	700	Ilahi, I. et al. (2020)	Urdu	8,092	40,460
Generative image captioning in Urdu using deep learning [28]	Flickr8k	1800	Afzal, M. et al. (2023)	Urdu	8,092	40,460
Deep Learning-Based Urdu Image Captioning [29]	Flickr8k	8000	Humayun S. et al. (2024)	Urdu	8,092	40,460
Automatic Textual Description Generation of Natural Images using DNN [30]	Own Dataset	999	Sidra S. et al. (2017)	Urdu/ English	999	999

So, in this paper, the Flicker30k dataset was used, which is an extended version of Flicker8k that can be translated into Urdu. Our model uses two deep learning architectures. ResNet-50 and NASNetLarge, as encoders to extract key visual features from images. These features are then processed by an LSTM (Long Short-Term Memory) [31] Network, which serves as the decoder to generate captions. The encoder extracts features from images, and then these features and vector files originating from captions are passed to a decoder. The decoder generates a caption using a greedy search approach in sequence. All these generated captions are evaluated using evaluation metrics which are BLEU and its variant, and achieve effective outcomes.

Despite its significance, Urdu remains an under-resourced language, with complex grammar and morphology[32]. Additionally, there are difficulties in text tokenization and language modeling and a lack of data available for Urdu over the internet. It is necessary to expand resources and solutions for Urdu to promote research in the language. Especially when the benchmark corpora have been established for almost all English research problems, Urdu remains an under-resourced language. This poses a significant challenge for developing an Urdu image captioning system. There are several challenges associated with the generation of captions in Urdu. Some of them are:

(1) Lack of a sizeable dataset in Urdu for the development, comparison, and evaluation of deep learning and transfer learning strategies for creating captions from images in Urdu.
(2) The previous model provided limited technical results for accurate prediction, primarily relying on score metrics.
(3) There is also a need for a robust model that generates the caption of the given image in Urdu language. There is no large-scale and reliable model available for generating Urdu captions from images.

The contribution of this work is given below:

(1) In this paper, a new dataset for image captioning in Urdu is introduced, leveraging the well-known Flickr30k dataset. The Urdu image captioning dataset UC-23-RY comprises 31,783 images, each accompanied by multiple captions, resulting in a total of 158,915 Urdu captions. These captions were generated through a semi-automated translation approach. The creation of UC-23-RY addresses a critical gap in Urdu language resources and provides a robust resource for training and evaluating image captioning models.
(2) An approach using deep learning-based methods is proposed, ResNet-50-LSTM and NASNetLarge-LSTM, for Urdu image captioning. This approach is designed to effectively capture and integrate visual and textual features, facilitating the generation of coherent and contextually relevant captions in Urdu.
(3) An extensive evaluation of the developed models was conducted using the BLEU score metric and its variants to assess the quality of the generated captions. The ResNet-50-LSTM and NASNetLarge-LSTM models demonstrated superior performance compared to state-of-the-art methods.

The rest of the paper is structured as follows: The Related Work section discusses previously developed datasets and methods. The Corpus Generation Process section describes the construction of the UC-23-RY dataset. The Materials and Methods section explains the implementation workflow and details the proposed approach. The Experimental Results section presents and analyzes the outcomes of the experiments conducted. The Discussion and Limitations section provides an evaluation of the findings and limitations of the study. Finally, the Conclusion and Future Work section summarizes the key findings of the study and suggests potential directions for future research.

## Related Work

**Fig 3:**
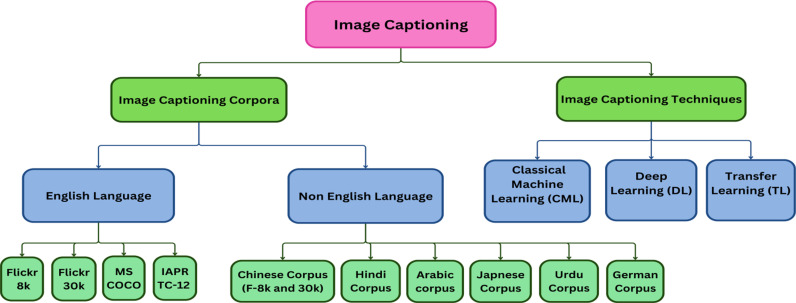
Image Captioning Taxonomy: An Overview of Corpora and Techniques.

The literature can be divided differently, but here literature is subdivided into image captioning corpora and techniques used for image captioning. Then, this subdivision of literature is also further divided into sub-parts. More details of this subdivision are provided in the following paragraphs and illustrated in Fig 3.

### Image Captioning Corpora

Existing corpora used for captions for images can be separated into two main subcategories as shown in Fig 3:

(1) Captioning corpora for the English language.
(2) Captioning corpora for non-English language.

#### Captioning Corpora for the English Language.

The majority of image captioning research has been conducted using the English language. The following image captioning corpora for the English language have been constructed from different studies and are discussed below and summarized in Table 2.

**Table 2:** English language corpora.

Dataset	Language	Images	Captions
Flicker 8K	English	8,092	40,460
Flicker 30K	English	31,783	158,915
MS COCO	English	164,062(MS COCO 14) 328,124(MS COCO 15)	820,310(MS COCO 14) 1,640,620(MS COCO 15)
IAPR TC-12 corpus	English	20,000	20,000

##### Flickr-8k Corpus

In 2013, Hodosh. et al. [12] provided two datasets in English to develop retrieval-based image captioning. The Flickr8k dataset has been extensively utilized in studies [33–37] to advance the state of art in image captioning. To enhance the accuracy of image captioning, modern techniques such as Convolutional Neural Networks (CNNs), Recurrent Neural Networks (RNNs), Long Short-Term Memory (LSTM) networks, Attention Mechanisms, and other approaches have been employed. Consequently, over time, image captioning models have performed noticeably better, contributing to the development of various real-world applications such as image tagging, retrieval, and description.

##### Flickr 30k Corpus

In 2015, Plummer. et al. [14] developed the Flick-30k corpus as a benchmark for Automatic Image Captioning. It was an extension of the Flick-8k corpus and contained 31,783 images, each with five annotations on the captions. The annotations were highly structured, but due to the complexity of a large variety of images, ambiguity in object identification, and unrealistic crowdsourcing annotations, the authors proposed a multi-stage pipeline technique for the annotation process. According to the aggregated information gathered during the annotation process across all five captions, humans, clothing, body parts, vehicles, instruments, and various other objects were identified in 94.2%, 12.0%, 69.9%, 28.0%, 13.8%, 4.3%, and 91.8% of the images, respectively. Flicker 30K is used by various papers for image captioning for making captioning models using part of a speech guidance module [38], a dual-model transformer for image captioning [39], and image caption generation with a caption-to-speech mechanism [40].

##### IAPR TC-12 Corpus

The IAPR TC-12 Corpus [41] comprised 20,000 images, each with at least one English sentence, and was extracted from a private collection of photographic pictures. The annotation guidelines allowed for words in English, Spanish, and Portuguese. This image collection was created for the Image CLEF (CLEF Cross-Language Image Retrieval Track). The TC-12 benchmark for IAPR (Image Retrieval benchmark) was established as a result.

##### MS COCO Corpus

MS COCO [15] (Microsoft Common Objects in Context) is a popular dataset for computer vision tasks involving object detection and captioning. The MS COCO 15 collection comprises 330,000 images, each with a half-million caption labels. The images in MS COCO belong to 80 different classes, such as individuals, creatures, automobiles, and domestic items. The MS COCO 14 collection consists of 164,062 images with 995,684 captions. MS COCO has been utilized by numerous researchers in their studies to create advanced models for image captioning [42–49].

#### Captioning Corpora for Non-English Language.

Most of the existing corpora are in English, leading to a lack of diversity in other languages. Many language corpora developed for image captioning are simply translated versions of the popular English-based Flickr-8k corpus. The Flickr30k dataset is primarily available in English, with limited translations into other languages [50]. The dataset includes 31,783 images, each with five English captions. While researchers may have translated these captions into other languages for their experiments, such translations are not part of the official Flickr30k dataset. Each dataset with several images and their caption details is mentioned in Table 3.

**Table 3:** Non-English language corpus.

Dataset	Language	Images	Captions
Flickr8k-CN	Chinese	8,092	40,460
Flicker 8k Hindi Corpus	Hindi	8,092	40,460
Flickr8k Urdu Corpus	Urdu	8,092	40,460
Arabic Corpus (Flicker 8k, MS COCO)	Arabic	8,092 328,124(MS COCO 15)	40,460 1,640,620(MS COCO 15)
F30Kent-JP	Japanese	31,783	158,915
Flicker 30k CN Corpus	Chinese	31,783	158,915
Multi30K	German	31,783	158,915

##### Chinese Language Corpus (F-8k)

In 2016, a bilingual version of the Flickr-8k [51] Chinese corpus was developed, known as the Flick-8k CN corpus, which consisted of Chinese captions. To create the Chinese captions, the authors used machine translation tools such as Google and Baidu. For example, “black” appeared only 116 times in Chinese captions compared to 3,832 times in English captions.

##### Hindi Language Corpus

In 2020, Rathi et al. developed the Flick-8k-Hindi corpus [52] by using the Flick-8k English corpus as the base corpus. Due to budget and time constraints, they opted to use a machine translation approach to construct the Hindi edition of Flick-8k. They used the Google Cloud Translator API, which offers free translation to approximately more than 100 languages.

##### Arabic Language Corpus

In 2018, Al-Muzaini et al. [6] developed an Arabic image-captioning corpus by combining portions of both the Flickr-8k [12] and MS COCO corpora [15]. In total, the Arabic corpus included 3,247 images with 16,663 Arabic captions and 9,854 words in the vocabulary. The lengthiest caption in the corpus consisted of 27 words.

##### Japanese Language Corpus

In 2020, Nakayama et al. [53], developed a Japanese corpus from Flicker 30k [14]. The study presents a multilingual multimodal corpus, named Flickr30k Entities JP (F30kEnt-JP), by extending the original Flickr30K Entities dataset with Japanese translations.This is the first bilingual image caption dataset with captions in two languages at the same time. The study investigated the worth of new Japanese labeling through phrase localization experiments and found improved performance.

##### Chinese Language Corpus (F-30k)

In 2017, Lan Weiyu et al. [54] created a bilingual version of the Flickr-30k [14] English corpus, known as the Flick-30k CN corpus, which consisted of Chinese captions. To create the Chinese captions, the authors used machine translation tools such as Google and Baidu. The Flick-30k CN corpus includes both machine-translated and human-generated Chinese captions, with the latter being collected through crowdsourcing.

##### German Language Corpus

In 2016 Elliott and Desmond [55] proposed the creation of the Multi30K benchmark to promote research in bilingual multimodal capabilities beyond the English language. The German translations by expert translators differ from the original crowed-sourced English description. The dataset expands upon the Flickr30k dataset. The dataset is useful for various tasks, including multilingual image description and multimodal machine translation.

##### Urdu Language Corpus

In 2020, Ilahi, I. et al. [27] prepared a dataset in the Urdu language by translating the Flickr8k dataset. Specific syntactical and grammatical norms for the Urdu language were followed to generate the dataset. Following that, Afzal, M. K., et al. [28] improved the dataset and made it publicly available for use in 2023.

### Image Captioning Techniques

With the impressive advancements in computer vision, several techniques have been created that have achieved remarkable success in Image Captioning and set new benchmarks.

There are three approaches, as shown in Fig 3, currently being utilized in the development of image captioning systems:

(1) Classical Machine Learning (CML)
(2) Deep Learning (DL)
(3) Transfer Learning (TL)

#### Classical Machine Learning.

Classical Machine Learning (CML) [56] is a traditional approach to machine learning that has been in use for several decades. CML models are typically based on handcrafted features designed to capture specific aspects of the images, for example, hue, feel, and form. These attributes are used as input to machine language algorithms, such as Support Vector Machines (SVMs) [57] or Random Forests [58], to learn from the data and generate new captions.

In 2012, Mitchell et al. [59] utilized contextual and syntactical techniques to produce English captions. They employed a template-based technique, whereby the syntactic trees provided information about the image’s visual content as interpreted by the computer. Mason et al. [60] introduced a non-parametric density estimation method for image captioning in 2014. They utilized the SBU-Flickr dataset, which includes defined labels. The non-parametric density estimation method involved assigning the same label word to an unseen image that had similar contextual characteristics as the seen images. The scores of existing sentences on the query image were calculated using a word probability distribution method, and the sentence with the highest score was selected. In 2013, Hodosh et al. [12] created two English language corpora to develop retrieval-based IC. They utilized the retrieval-based technique, which involved slecting captions from a pre-existing pool of sentences. In their proposed method, ranking was implemented.

#### Deep Learning.

In 2024, Humayun S. et al. [29] proposed a caption generation model for the Urdu language by applying deep learning techniques like InceptionV3 and LSTM to translate the Flickr-8k dataset into Urdu, with impressive outcomes. In 2017, Sidra [30] suggested a deep neural network-based automatic textual description model for the Urdu Language based on 999 natural images with 999 captions in both Urdu and English. In 2014, Socher et al. [61] incorporated both retrieval-based and template-based techniques in IC using deep learning (DL). They employed the English-annotated Amazon Mechanical Turk Dataset [62], which consisted of 1000 images with five English captions per image. In 2016, Wang et al. [63] used deep learning to caption images and utilized the Flick-8k English corpus. They proposed a unique method by merging both RNN and LSTM architectures in parallel. In 2014, Kiros et al. [64] introduced a multimodal neural approach for language modeling by proposing a neural implementation. In 2015, J. Mao et al. [65] utilized several English corpora to propose a multimodal RNN (m-RNN) model for generating novel English captions. In 2015, Vinyals et al. [66] aimed to apply deep neural machine translation to image captioning and used an LSTM in an RNN to serve as a decoder to provide a caption after CNN had encoded the provided image. In 2018, Shaung et al. [67] conducted a comprehensive survey of research in image captioning and noted that RNNs suffer from vanishing gradient issues. This approach prioritizes the most salient words in generating the name and relationship of significant objects within the image. In 2019, a classification of IC methods based on different attributes was presented by Z. Hossain [68]. In 2018, Srinivasan et al. proposed a hybrid system [69] that builds complete phrases utilizing the general keywords by applying a Long Short-Term Memory (LSTM) to a multilayer CNN to generate vocabulary describing images. In 2015, compositional image captioning was planned by Fang et al [70], which had independent functional units. The visual models focused on the image’s visual content and passed along the feature to the language model, which then composed the caption. In another approach, Mao et al. [71] proposed a novel object-based EIC method. Aneja et al. [72], in 2018, developed a CNN-based module that used masked convolutions instead of RNN and LSTM due to the issue of vanishing gradients. In 2020, Rathi [52] introduced deep learning methods to the Flick-8k Hindi dataset. The model was trained using a CNN-LSTM encoder-decoder architecture. Castro investigated the influence of different hyperparameter arrangements [73] for image captioning tasks in the field of computer vision research, using an encoder-decoder visual attention framework.

#### Transfer Learning.

In 2020, Wang et al. [74] proposed a cross-lingual approach in which they added an independent recurrent structure at the caption generation stage by utilizing the Flick-8k [12] English dataset and its Chinese counterpart, Flickr-8k CN [51]. Degadwala [75] utilized transfer learning for Image Captioning in 2021. To extract image features, they used a pre-trained inception-V3 model and added a fully linked layer. In 2019, Perdana et al. suggested a method for cross-domain image captioning termed Multimodal instance-based Deep Transfer Learning (MIBTL). They performed instance-based transfer learning to identify which data improved the training process of the target domain dataset [76]. In 2022 Ayoub et al. suggested a methodology for automatically creating captions for images [77] that integrates pre-trained CNN, the Bahdanau attention mechanism, and transfer learning approaches to predict image captions. Their study compared the performance of VGG16 and InceptionV3, two pre-trained CNNs. In 2021, Banerjee et al. introduced a model for annotating images using transfer learning [78] that given a target dataset with a small number of style-based ground-truth captions, produces style-based captions. After training on the source dataset, the model is adjusted to provide style-based captions for the target dataset. The model is presently being tested as proof of concept at Myntra [79] to gather user feedback, with the potential to enhance the customer experience and increase the add-to-cart ratio by providing additional style-based captions for fashion apparel.

## Corpus Generation Process (UC-23-RY development)

Our main objective was to create a sizeable benchmark corpus for the Urdu Language as part of research in MUST (Mirpur University of Science and Technology). The steps involved in creating a dataset are described below:

### Data Collection

The initial import images for the development of the dataset were collected from Flickr30k [80] for Urdu image captioning. For each image, five Urdu sentences are written as part of multiple captions resulting in 158916 captions and 31783 images. It is called the Urdu Captioning dataset (UC-23-RY).

### Data Cleaning and Pre-Processing

For processing by computer AI model, Urdu captions were cleaned up by removing punctuation marks and special characters. In the next step, Urdu captions were tokenized to generate vocabulary and identify correct word limitations. After tokenization, 18,804 unique words were obtained. Following preprocessing and data cleaning 158,915 captions are assembled.

### Sentences Creation Process and Guidelines for UC-23-RY Corpus

For sentence creation, both automatic and manual methods were used for the sentence writing process.

#### Automatic Translation.

Initially, the English captions were first translated into Urdu using the Bulk translator tool [81] due to its ability to handle large volumes of text. However, some limitations were observed:

- **Contextual Errors:** Automatic translation tools lack contextual knowledge, leading to inaccurate translations.
- **Grammatical Errors:** The difference between English and Urdu grammar led to errors that automatic tools could not resolve.
- **Limited Resources for Urdu:** As Urdu is an under-resourced language, Bulk translators struggled with maintaining quality and consistency across translation.

#### Manual Inspection and Correction.

Due to errors in automatic translation, manual inspection and correction were performed on the translated Urdu text. An Urdu language expert was tasked with:

- Ensuring translation matched the contextual meaning of the original English captions.
- Correcting any grammatical errors and improving sentence fluency to maintain semantic accuracy.
- Ensuring consistent terminology across the dataset

#### Challenges.

The translation and correction process were time-consuming, with over 158,000 captions requiring attention. This process only took 2–3 months, largely due to the need for manual inspection and the limitations of translation tools.

### Rounds of Quality Control

#### Initial Automatic Translation.

- The initial translation of English captions into Urdu was performed using online tools such as Google Translator and Bulk Translator.
- Bulk translator was chosen due to its ability to handle large datasets, but it faced limitations in accuracy and context preservation.

#### Manual Inspection and Correction.

After automatic translation, a round of manual inspection and correction was conducted, focusing on the testing data. In contrast, we performed this process on the entire dataset to rectify grammatical and contextual errors.

#### Final Review.

The final review involves ensuring the accuracy of the corrected captions. Special attention was given to maintaining syntactic and semantic alignment between the original English captions and their Urdu translations.

### Translation Quality Measurement

#### Human Evaluation.

A linguistic expert manually reviewed and corrected the automatically translated captions. The focus was on grammatical correctness, fluency, and contextual accuracy.

#### Error Correction Based on Observations.

A subset of captions with significant translation errors was identified during manual inspection. This phase ensured that any inaccuracies were fixed, particularly in critical testing data.

#### Consistency Check.

Throughout the process, consistency in terminology and translation style was maintained. Continuous observation ensured that corrections followed the guidelines established for the UC-23-RY corpus.

### Corpus Normalization

The UC-23-RY corpus is composed of 31,785 images and 158,915 Urdu captions. The corpus has been normalized in the CSV format, which is manually corrected and inspected to facilitate a comparison between automatic and manual translation as shown in Table 4.

**Table 4:** Manual Inspection and Correction.

Instance Number	Flicker 30k caption (English)	Flicker 30k Automatic caption (Urdu)	Manually corrected Flicker 30k Caption (Urdu)
**Image No: 2903469015.jpg (Flickr 30K)**	Two greyhounds with numbers run in a race on a track.	نمبروں کے ساتھ دو گرے ہاؤنڈ ایک ٹریک پر ریس میں دوڑتے ہیں۔	دو گرے ہاؤنڈ جن پرنمبرہیں ایک ٹریک پر ریس میں دوڑ رے ہیں۔
	Two muzzled greyhounds’ dogs racing around a track.	دو مسلط گرے ہاؤنڈ کتے ایک ٹریک کے گرد دوڑ رہے ہیں۔	دومنہ بند باندھے گرے ہاؤنڈ کتے ایک ٹریک پر دوڑ رہے ہیں۔
	Muzzled greyhounds are racing along a dog track.	مسلئے ہوئے گرے ہاؤنڈ کتے کے راستے پر دوڑ رہے ہیں۔	منہ بندھے گرے ہاؤنڈ کتے دوڑ کے میدان میں تیزی سے دوڑ رہے ہیں۔
	A greyhound wearing a yellow jacket runs a race.	پیلے رنگ کی جیکٹ پہنے ایک گرے ہاؤنڈ ریس چلا رہا ہے۔	پیلے رنگ کی جیکٹ پہنے ایک گرے ہاؤنڈ ریس میں دوڑ رہا ہے۔
	A greyhound wearing number 8 is racing.	ایک گرے ہاؤنڈ نمبر 8 پہنے ہوئے ریسنگ کر رہا ہے۔	ایک گرے ہاؤنڈ نمبر آٹھ کی وردی پہنے ہوئے دوڑ رہا ہے۔

### Corpus Characteristics

**Fig 4:**
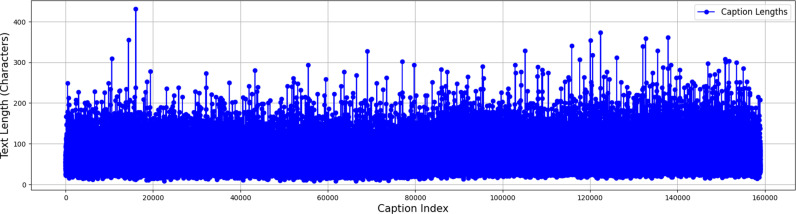
Distribution of caption lengths (index vs text length).

Table 5 summarizes the key characteristics of the dataset. The entire dataset has been used, which includes 31,783 images, each with an average of five English captions per image. A native speaker of Urdu translated the captions by hand, and then another native Urdu speaker went through several rounds of quality control. The UC-23-RY dataset includes a total of 18,804 unique words and a maximum caption length of 431 words as shown in Fig 4, which shows the distribution of text lengths for each caption in the dataset. The x-axis corresponds to total number of captions, while the y-axis indicates the length of caption in term of the number of words or characters. The visualization helps in understanding the variation in caption length across the dataset.

**Table 5:** UC-23-RY Corps Characteristics.

Metric	Count
**Total number of UC-23 Images**	31,785
**Total number of UC-23 Captions**	158,915
**Number of UC-23 Caption per Image**	05
**UC-23 Training Images**	25,426
**UC-23 Testing Images**	6,357
**UC-23 Training Image Captions**	127,130
**UC-23 Testing Image Captions**	31,785
**Maximum Length of UC-23 Images caption**	431
**Total number of unique words/vocabularies**	18,804

## Materials and Methods

**Fig 5:**
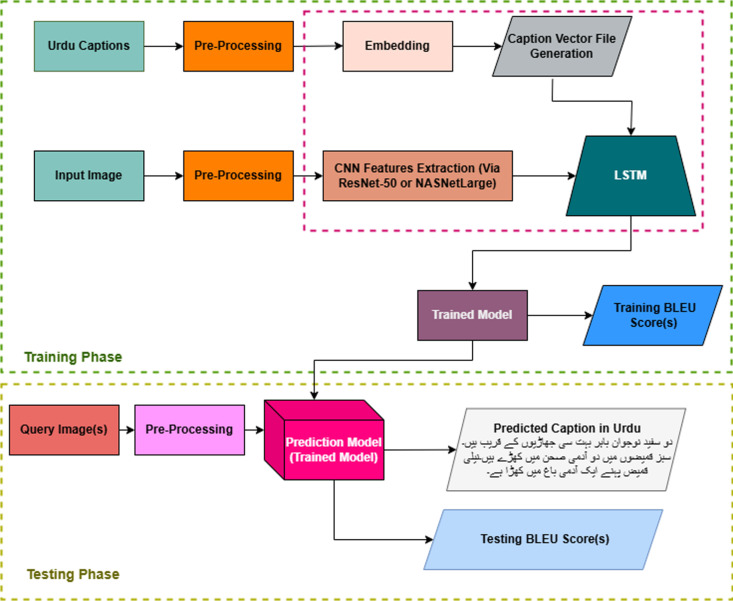
Flowchart of Urdu image caption generation process.

An encoder-decoder mechanism is adopted for automatic image captioning, where ResNet-50 [82] and NASNetLarge [83] act as encoders and LSTM [31] acts as a decoder. ResNet-50 or NASNetLarge utilize pre-trained ImageNet weights for feature extraction from visual data. LSTM is a more progressive type of RNN that can remember long-term dependency, so it gives the sequence prediction for query images as shown in Fig 5.

### Implementation Workflow

This Pseudocode outlines the steps as shown in Fig 4 involved in generating Urdu captions for images using a deep learning model that integrates CNN features extraction (via ResNet-50 or NASNetLarge) and LSTM sequence modeling.

**Inputs** Image dataset (UC-23-RY)

**Outputs** Urdu captions for input images.

a. **Preprocess Stage**

- Resize images and normalize pixel values.
- Tokenization and Preprocess Urdu captions.
- Apply word embedding to Urdu captions.

b. **Visual Feature Extraction Stage**

- Use CNN (ResNet-50 or NASNetLarge) to visual features from input images.
- Train model for n epochs.
- Save the trained model and monitor its performance.

c. **Caption Generation Stage**

- Extract image features from CNN (ResNet-50 and NASNetLarge) and feed them to LSTM.
- Train the LSTM model with image features and tokenized captions sequences.
- Update LSTM hidden states in both forward and backward direction to generate the sequence of words for captions.
- Optimize LSTM hyperparameters during training.
- Convert the generated caption sequence to Urdu text using word embedding and obtain the predicted caption for an input image.

d. **Testing phase**

- Evaluate the trained model by passing query images.
- Computer performance metrics (BLEU Scores).
- Compare predictions to assess the accuracy.
- Analyze results to fine-tune model parameters and improved performance.

### Dataset

For the experiment presented in this paper, the entire UC-23-RY dataset was used (containing 158915 Urdu captions along with 31,785 images). A train set comprising 25,426 images (approximately 80%) and a test set of 6,357 images (approximately 20%) were randomly created from the dataset. There are five captions for each image, which yields a split of 127,130 train and 31,785 test captions. The experiment uses the remaining 6,357 test images after the model has been trained on 25,426 training images as shown in Table 6.

**Table 6:** Division of training and testing images based on a split ratio.

Language	Dataset	Split Ratio	Training split	Testing split	Total Images
Urdu	UC-23-RY	80:20	25,426	6,357	31,785

### Image and Textural Features Extraction

Regarding our developed model, ResNet-50 and NASNetLarge are used separately, not together, for feature extraction in different experiments. The input image size fed into the network is 224 by 224 pixels for ResNet-50 and 331 by 331 for NASNetLarge. ResNet-50 and NASNetLarge were trained on the ImageNet dataset, which contains over a million images spanning 1000 object classes, such as animals, pencils, keyboards, and more. Utilizing the pre-defined ImageNet weights of ResNet-50 and NASNetLarge for the problem constitutes transfer learning. The final layer is a fully connected (dense) layer, a prediction that can be removed to bind the image feature vector of the Urdu caption. For textural features, Urdu word embedding that contains Urdu tokens with 300-dimensional vectors was utilized.

### Multimodal Approach

**Fig 6:**
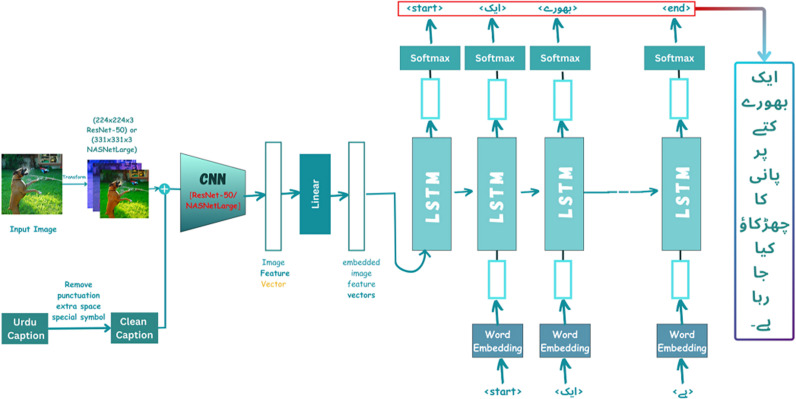
Proposed approach for Urdu image captioning.


In the proposed multimodal approach, illustrated in Fig 6, visual and textual data are combined to generate Urdu image captions, hence it is referred to as multimodal. The image is processed through two different CNN models, ResNet-50 or NASNetLarge, which are used independently with LSTM as a decoder. ResNet-50 extracts image features using a 50-layer deep network, while NASNetLarge offers more complex feature extraction due to its large architecture and capability to generalize across a wide range of objects. The extracted image features are converted into vectors, which are then passed to LSTM to generate captions word by word. The fusing process in our model is achieved through a concatenation process, where the image embedding vector and caption/text embedding vector are combined along a specific dimension to form a single input vector. Mathematically, if **I** represent the image embedding vector of dimension d_1_ and **T** represents the caption/text embedding vector of dimension d_2_, the fuse vector **F** in Eqs 1 is defined as:

F = [I; T] (1)

where **[*I; T*]** denotes the concatenation of vectors **I** and **T**. This fuse vector **F** is then passed to subsequent layers, including CNN and LSTM for processing. Both models were chosen for their strong performance in feature extraction tasks, with ResNet-50 providing efficient processing and NASNetLarge offering detailed feature extraction, ensuring robust caption generation across various contexts.

#### Resnet-50-LSTM Based Captioning Model.

For the language model (ResNet-50-LSTM), the input shape is 2048 with a dimension of 256 images, a maximum length of 431, and a vocabulary size of 4732 after removing low-frequency words with 300 embedding dimensions of text passed to the LSTM model with a dropout rate of 0.3. A fully connected neural layer with cell spatial size (256) and hidden layer (256) is used. This dense neural network is used for each name vector a_i_ which is based on the decoder’s last hidden state h_t-1_. The projected hidden space was concatenated with each input vector using summation, which additionally produced a ReLU-activated shape (4732,256). The vector is used to participate in the (2048, 300) shape vector, which results in the context vector representation of the visual feature. LSTM needs fixed-length sequences, so varying sentence lengths are padded to match the maximum size of 86. This uniformity allows for consistent processing in a dataset.

A 256-size custom embedding layer has been added, during training, and learns a fixed size length continuous domain representation. The LSTM decoder uses this as its final representation of words. The LSTM has a hidden space size of 256. To forecast the next word, an updated hidden space is employed, which is sampled by a fully connected layer. This projects 300 vectors of each word into the vocabulary. It is related to SoftMax for verbal prediction. Categorical_Crossentropy loss (multicategory) is used to backpropagate the gradient.

In the benchmark approach, the last prediction, S_t-1’_^s^ word embedding (256), is utilized as input for the subsequent time step. In the LSTM, the hidden state ht is updated at each cycle. The vector is then concatenated and fed into a single LSTM decoder, which produces the next word.

The Adam optimizer is utilized by default with 1e^-3^ learning. According to the trainer, the dropout value of 0.3 was used in 30% of randomly selected workouts. A training cycle is applied to a deep-learning model with 500 epochs and a group of size 5. The training data is fed into the model using a generator (data_generator) for each loop and the model is updated using the ‘fit_generator’ method with a specified number of 32 steps per epoch. Then, each trained file is saved with a unique name. If training stops at any stage due to any cause, then the next time it should be started from the last saved file where training is stopped.

Categorical cross-entropy loss and accuracy were tracked. The loss is continuously decreasing and accuracy improves after each epoch. After 500 epochs, the accuracy, which was 0.079 at the beginning, increased to 0.937, and the loss, which was 6.239 at the beginning, decreased to 0.139. The encoder is trained using transfer learning, with its weight frozen, while the decoder is trained only once. The first convolutional block would typically have learned low-level image processing features such as identifying lines, edges, and curves. Only the convolutional blocks for ResNet-50 are fine-tuned, while the initial block remains intact. As a result, the foundations remain the same as shown in Table 7, which displays the trained model’s hyperparameters.

**Table 7:** Hyper-parameter of the trained model.

	ResNet-50-LSTM	NASNetLarge
**Dataset**	Flickr30k-Urdu	Flickr30k-Urdu
**Trainable Parameter**	23,534,592	84,720,150
**Trainable LSTM Parameter**	2,376,828	2,376,828
**Training Images**	25,426	25,426
**Epochs**	500	700
**Batch Size**	05	05
**Steps**	32	32
**Optimizer**	Adam	Adam
**Learning Rate**	1e^-3^	1e^-3^
**Dropout**	0.3	0.3
**Accuracy**	0.93	0.94
**Loss**	0.13	0.12

### Nasnetlarge-LSTM based captioning model

For the language model (NASNetLarge-LSTM), the input shape is 4032 with a dimension of 256 for the image and a maximum length of 431. The vocabulary size is 4732 after removing low-frequency words with 300 embedding dimensions of text passed to the LSTM model with a dropout rate of 0.3. A fully connected neural layer with a cell spatial size (256) and a hidden layer (256). The dense neural network is utilized on individual name vectors a_i_, which are dependent on the final hidden state h_t-1_ of the decoder. The projected hidden space was concatenated with each input vector using summation, which additionally produced a ReLU-activated shape of (4732,256). To achieve the context vector representation of an image feature, the vector is subsequently utilized to participate in the shape vector (4032, 300). LSTM needs fixed-length sequences, so varying sentence lengths are padded to match the maximum size of 86. This uniformity allows for consistent processing in the dataset.

The LSTM decoder uses this as its final representation of the words. The LSTM has a hidden space size of 256. To forecast the next word, we utilize an updated hidden space sampled by a fully connected layer, which projects 300 vectors of each word into the vocabulary. This is related to SoftMax for verbal prediction. Categorical_cross-entropy loss (multicategory) is used to backpropagate the gradient.

In the benchmark model, the most recent prediction, S_t-1’_^s^ word embedding (256), is utilized exclusively as input for the subsequent time step. In the LSTM, the hidden state ht is updated at each cycle. The vector is concatenated and fed into a single LSTM decoder to generate the next word.

The Adam optimizer is utilized by default with 1e-^3^ learning. According to the trainer, the Dropout value of 0.3 was used in 30% of randomly selected workouts. A training cycle is applied to a deep-learning model with 700 epochs and a group of size 5. The train data is fed into the model using a generator (data_generator) for each loop and the model is updated using the ‘fit_generator’ method with a specified number of 32 steps per epoch.

Then, each trained file is saved with a unique name. If the training process is interrupted for any reason, it should resume the next time it starts from the most recently saved file. Categorical Cross-entropy loss and accuracy were tracked. The loss is continuously decreasing and accuracy improves after each epoch. After 700 epochs, the accuracy, which was 0.0806 at the beginning, increased to 0.9425, and the loss, which was 6.3577 at the beginning, decreased to 0.1218. Transfer learning is applied to the encoder, keeping its weights frozen, and only the decoder was trained. The first convolutional block would normally have learned low-level features that are image processing, including detecting lines, edges, curves, etc. Only the convolutional blocks for ResNet-50 are fine-tuned, while the initial block remains intact. As a result, the foundations remain the same as shown in Table 7, which displays the hyperparameters of the trained model.

### Computational Resources and Time Analysis

The experiment was conducted on a system with an Intel Core i5-6300HQ CPU @ 2.0 GHz, 8 GB of RAM, and a 64-bit operating system running on Windows. The deep learning model is implemented using Python and TensorFlow, with Keras as the high-level neural networks API. While the system supplies sufficient resources for model training and evaluation, the relatively modest hardware specifications necessitated careful management of computational resources, particularly in dealing with large datasets and more complex models like NASNetLarge. After the translation, the deep learning models are trained with careful monitoring of time spent on each epoch. The ResNet-50-LSTM model was trained for 500 epochs, and the NASNetLarge-LSTM model was trained for 700 epochs. The time trained per epoch varied based on the complexity of the model and training steps. The total time required to complete the training for each model is summarized in Table 8.

**Table 8:** Time elapsed during training of ResNet-50-LSTM and NASNetLarge-LSTM model.

Model	Epochs	Time Elapsed (seconds)	Steps Per Epochs	Total Time (seconds)
ResNet-50-LSTM	500	110-215s	3-7s/step	~1 hour 18 minutes
NASNetLarge-LSTM	700	52-210s	2-7s/step	~1 hour 33 minutes

In the case of ResNet-50-LSTM, the training of 500 epochs required a total of approximately 1 hour 18 minutes. For NASNetLarge-LSTM, the training of 700 epochs took roughly 1 hour 33 minutes. Both models showed varying time durations per epoch due to the difference in the number of steps and model complexity.

## Experimental Results

The experiment is conducted using multimodal approaches that combine image and text processing techniques. Our steps include translating English captions into Urdu with various models trained on different datasets.

We compute accuracy as a percentage-based measure derived from BLEU Scores to provide an intuitive representation of model performance. BLEU is a parameterized metric whose value varies with the change of parameters. It considers quantitative metrics to find how much a certain prediction is valid by giving some score.

Accuracy in Eqs 2 is calculated as:


Accuracy (\nonumber%) = BLEU Score x 100 (2)

The BLEU Score is based on the sequential conformance of N-gram precision with a brevity penalty to account for length differences, it ranges from 0 to 1 (or 0% to 100%), even though natural language contains significantly more flexible formulations where more flexible formulations where multiple words or their combinations may convey the same semantic idea. This results in automatically generating predictions using greedy search and the results are evaluated using metrics for BLEU-1, BLEU-2, BLEU-3, and BLEU-4, as shown in Fig 8.

**Fig 7:**
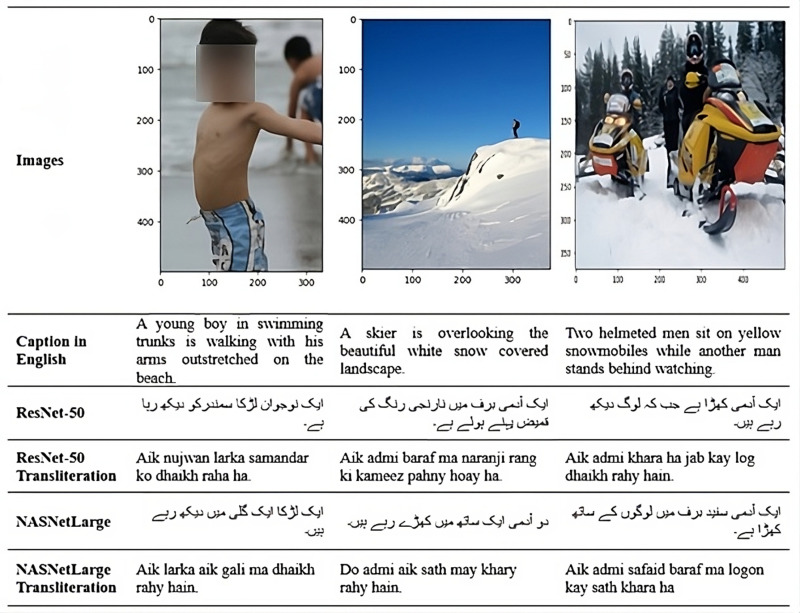
Captions in Urdu using ResNet-50 and NASNetLarge models with their transliterations.

**Fig 8:**
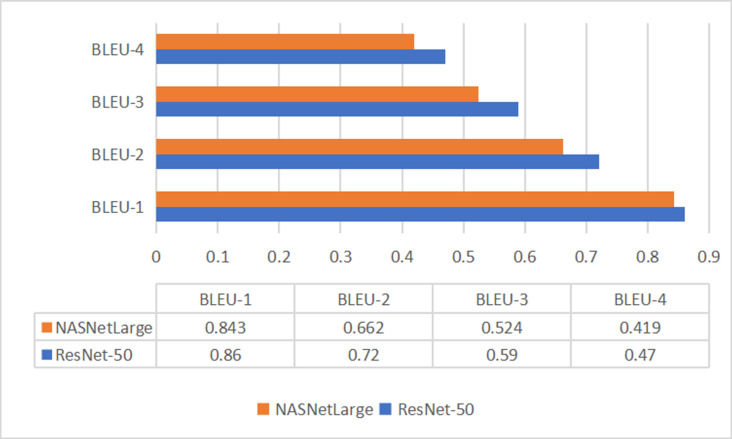
ResNet-50 and NASNetLarge results for BLEU – (1,2,3,4) Scores.

For comparison, models were examined using the Flickr8k dataset. Our model which uses ResNet-50 as the encoder and LSTM as the decoder with Flickr8k, achieved BLEU-1 scores of 75.3, BLEU-2 scores of 63.2, BLEU-3 scores of 50.9, and BLEU-4 scores of 40.2. In contrast with Flickr30k, our models show low performance. Using the more extensive Flickr30k dataset and ResNet-50, our model demonstrates improved performance and highlights its effectiveness over those benchmarks.

In Table 9, Dual Model Transformer [39], CNN-LSTM [52], and Chinese caption model [51] present a comparative analysis of our proposed result with those reported in prior research using different languages (English, Hindi, and Chinese), highlighting improved performance achieved by our methodology. In our study, the Flickr30k dataset is used to translate English captions to Urdu. Our approach features an LSTM decoder combined with CNN-based encoders: ResNet-50 and NASNetLarge. The ResNet-50-LSTM model achieved a BLEU-1 score of 86, while NASNetLarge achieved 84, significantly outperforming previous Urdu-specific models. This improvement highlights the effectiveness of our approach and the richness of the UC-23-RY dataset. Additionally, our model outperforms studies in other languages such as Hindi, and Chinese, establishing the robustness and generalization of our method.

**Table 9:** BLEU scores for different datasets in different languages.

Model	Dataset	Language	BLEU-1	BLEU-2	BLEU-3	BLEU-4
Dual Model Transformer [39]	MSCOCO	English	85.01	78.37	70.34	48.33
CNN-LSTM [52]	Flickr-8k	Hindi	58.44	40.00	27.00	12.00
Chinese caption model [51]	Flickr-8k	Chinese	63.3	31.3	7.2	---
Attention-driven inject model [27]	Flickr-8k	Urdu	83	----	---	---
Generative Image captioning [28]	Flickr-8k	Urdu	72.5	56.9	42.8	31.6
Deep Learning-Based Urdu Image Captioning [29]	Flickr-8k	Urdu	52.8	----	---	---
**Proposed Model**	**Flickr 8k**	**Urdu**	**75.3**	**63.2**	**50.9**	**40.2**
**Proposed (ResNet-50-LSTM)**	**UC-23-RY**	**Urdu**	**86**	**72**	**59**	**47**
**Proposed (NASNetLarge-LSTM)**	**UC-23-RY**	**Urdu**	**84**	**66**	**52**	**41.9**

The remaining models, such as the Attention-driven inject model [27], Generative Image captioning [28], and Deep Learning-Based Urdu Image Captioning [29], presents a comparison of the performance metrics between the same language and two models proposed in this research, emphasizing their strengths and differences. The proposed approach for an Urdu image captioning system uses deep transfer learning methods, which differ in feature extraction approaches. For deep learning, image features are extracted using the CNN model while LSTM generates sequence prediction. Pretrained Urdu word embeddings such as urduvec are used for textual embedding as shown in Table 10.

**Table 10:** Feature and textural extraction model with random weights and urdu vector for a multimodal approach.

Sr No.	Image Feature Extraction Model	Weights	Textural Feature Extraction Model	Word Embedding	Multimodal
1	CNN (ResNet-50)	Random Weights Initialization	LSTM	Urduvec	CNN (ResNet-50)-LSTM
2	CNN (NASNetLarge)	Random Weights Initialization	LSTM	Urduvec	CNN (NASNetLarge)LSTM

A total of 6357 Flickr30k images were tested. Our trained model was utilized, which is ResNet-50-LSTM and NASNetLarge-LSTM. The ResNet-50 model contains 23M parameters and NASNetLarge contains 84M parameters as shown in Table 11. Both models utilized our own generated Urdu embedding token file. Mostly captions generated by the model give proper context about what happened in the image as shown in Fig 7.

**Table 11:** Parameter of Resnet-50 and NASNetLarge.

CNN	Number of Parameters
ResNet-50	23,534,592
NASNetLarge	84,720,150

From the findings of deep learning models, it was observed that the BLEU-1 score of ResNet-50 outperformed that of the NASNetLarge-LSTM model. The ResNet-50-LSTM model obtained the highest BLEU-1 score of 0.86 compared to NASNetLarge-LSTM with a 0.843 BLEU-1 score. A pre-trained CNN model was utilized, which has ImageNet weights with fine-tuning of the parameters according to our needs and is utilized for image feature extraction with a sequence model RNN used for processing textual data.

## Discussion and Limitation

The UC-23-RY dataset and the application of deep learning models, including ResNet-50-LSTM and NASNetLarge-LSTM, represent notable contributions to Urdu image captioning. In our study, the accuracy of the model was evaluated through a combination of automated and manual validation methods. The captions generated by the model were manually validated by both native and non-native speakers to ensure linguistic fluency, contextual accuracy, and naturalness. This manual review was conducted in multiple rounds to maintain consistency. Additionally, the caption was cross-verified with high-quality reference images and benchmarks to ensure their relevancy and alignment with ground truth. For automated evaluation, BLEU Scores (BLEU-1, BLEU-2, BLEU-3, BLEU-4) were used to measure the accuracy of predicted captions. The achieved BLEU-1 scores validate the effectiveness of these models in generating high-quality captions. By combining BLEU (1,2,3,4) scores for automated evaluation, manual checks for linguistic accuracy, and cross-validation with high-quality benchmarks, we ensured a comprehensive and reliable validation of the model’s performance. However, the study highlights several areas for improvement. Expanding the dataset and exploring the additional evaluation metrics could address limitations and enhance model performance. Future research should focus on increasing dataset size, integrating advanced language models, and optimizing computational efficiency to further advance Urdu image captioning. Regarding limitations, adding more images and their corresponding captions can increase linguistic diversity and enhance the ability of the model to produce captions for a variety of images. To compare and assess the models with huge datasets, additional robust models with different evaluation metrics might be employed.

## Conclusion and Future Work

Urdu captioning is a task that includes producing an appropriate caption in Urdu language. Although Urdu lacks resources, there is no benchmark corpus available for Urdu image captioning. Therefore, we develop the UC-23-RY corpus from the Flickr30k dataset, for Urdu image captioning. A semi-automatic translation approach was adopted. This is the first research that generated an Urdu dataset using Flickr30k images, prior most of the work was done on 8k with the mostly limited chosen images. This research also focuses on applying cutting-edge deep learning models that used CNN for images and LSTM for hybrid feature learning with the best BLEU-1 score of 0.86 for ResNet-50 and 0.82 for NASNetLarge.

Throughout our research, we identified some main development areas for image captioning that will be expanded upon in the future based on the study we conducted:

- Expanding the size of the UC-23-RY corpus by including other corpora such as MS COCO.
- Implementing advanced transformer models such as BERT, GPT, and T5 as language models to capture the contextual relationships in caption generation tasks.
